# Recent Updates on the Involvement of PI3K/AKT/mTOR Molecular Cascade in the Pathogenesis of Hyperproliferative Skin Disorders

**DOI:** 10.3389/fmed.2021.665647

**Published:** 2021-04-30

**Authors:** Laura Mercurio, Cristina Albanesi, Stefania Madonna

**Affiliations:** Laboratory of Experimental Immunology, Istituto Dermopatico dell'Immacolata, IDI-IRCCS, Rome, Italy

**Keywords:** PI3K, AKT, non-melanoma skin cancer, psoriasis, atopic dermatitis, hyperproliferation, apoptosis

## Abstract

PhosphoInositide-3 Kinase (PI3K) represents a family of different classes of kinases which control multiple biological processes in mammalian cells, such as cell growth, proliferation, and survival. Class IA PI3Ks, the main regulators of proliferative signals, consists of a catalytic subunit (α, β, δ) that binds p85 regulatory subunit and mediates activation of AKT and mammalian Target Of Rapamycin (mTOR) pathways and regulation of downstream effectors. Dysregulation of PI3K/AKT/mTOR pathway in skin contributes to several pathological conditions characterized by uncontrolled proliferation, including skin cancers, psoriasis, and atopic dermatitis (AD). Among cutaneous cancers, basal cell carcinoma (BCC) and cutaneous squamous cell carcinoma (cSCC) display PI3K/AKT/mTOR signaling hyperactivation, implicated in hyperproliferation, and tumorigenesis, as well as in resistance to apoptosis. Upregulation of mTOR signaling proteins has also been reported in psoriasis, in association with enhanced proliferation, defective keratinocyte differentiation, senescence-like growth arrest, and resistance to apoptosis, accounting for major parts of the overall disease phenotypes. On the contrary, PI3K/AKT/mTOR role in AD is less characterized, even though recent evidence demonstrates the relevant function for mTOR pathway in the regulation of epidermal barrier formation and stratification. In this review, we provide the most recent updates on the role and function of PI3K/AKT/mTOR molecular axis in the pathogenesis of different hyperproliferative skin disorders, and highlights on the current status of preclinical and clinical studies on PI3K-targeted therapies.

## Introduction

Phosphatidylinositol 3-kinase (PI3K) represents a family of kinases which play vital roles in mammalian cells by regulating proliferation, growth, and survival initiated by many growth and survival factors (1, 2). Dysregulation of PI3K-dependent signaling and, in particular, of PI3K/AKT/mammalian target of rapamycin (mTOR) pathway has been observed in different pathological conditions characterized by uncontrolled proliferation, loss of cell growth control, and decreased apoptosis. Aberrant PI3K/AKT/mTOR signaling is also observed in pathological skin, in particular in cutaneous cancer, as well as in chronic inflammatory diseases, such as psoriasis and atopic dermatitis (AD).

Among skin tumors, non-melanoma skin cancers (NMSC) refer to keratinocyte carcinomas and are classified into two major groups, namely basal cell carcinoma (BCC) and cutaneous squamous cell carcinoma (cSCC). BCCs are the most common human skin cancers, comprising about 75–80% of all skin tumors, and originate mainly from basal cell layer of epidermis (3, 4). cSCCs, less frequent, arise from squamous cells of epidermis and hair follicle stem cells (5), but they can also originate from dysplastic epidermal areas known as actinic keratoses (AK) (6). cSCCs are more dangerous and aggressive than BCCs, being able to invade and metastasize the dermis and local lymph nodes (6). In these pathological contexts, PI3K/AKT/mTOR signaling is hyperactivated and implicated in hyperproliferation and tumorigenesis, as well as in resistance to apoptosis (7, 8). PI3K signaling is mostly activated in the epidermal compartments, specifically in keratinocytes, following their exposure to environmental agents determining DNA alterations, such as ultraviolet (UV) radiation, and/or to cytokines aberrantly produced by activated immune cells (9).

The immune-mediated skin diseases psoriasis and AD can be both considered as hyperproliferative disorders in which epidermal keratinocytes respond to T lymphocyte-derived cytokines by altering growth, proliferation, and differentiation responses, accounting for major parts of the overall disease phenotypes (10–12).

Psoriasis is a chronic inflammatory skin disorder, in which skin-infiltrating T-helper (Th1, Th17, and Th22) lymphocytes promote keratinocyte hyperproliferation and terminal differentiation by releasing the pro-inflammatory cytokines IL-17A, IL-22, TNF-α, and IFN-γ (12–15). IL-36 cytokines released by keratinocytes themselves also determine impaired keratinocyte maturation and cornification in psoriasis (16–19). In addition, these cytokines upregulate PI3K/AKT/mTOR pathway, which in turn controls secretion of pro-inflammatory mediators by keratinocytes (20), enhances proliferation and impairs keratinocyte differentiation in skin affected by psoriasis (21).

The role of PI3K/AKT/mTOR in AD is less characterized than in psoriasis. AD is an immune-mediated skin disease characterized by alterations of skin barrier primarily due to loss-of-function filaggrin (FLG) mutations (22). During the acute phase of AD, inflammatory infiltrate is mainly represented by Th2 lymphocytes releasing type-2 cytokines, such as IL-4 and IL-13 (23, 24), which impair keratinocyte terminal differentiation and proper epidermal stratification (25, 26). In chronic AD, lichenified lesions appear, typically exhibiting altered epidermal hyperplasia, parakeratosis, and hyperkeratosis with amplification of Th2 axis and concomitant presence of Th1 cells releasing IFN-γ and TNF-α (27, 28). Recent evidence demonstrates the relevant role for mTOR pathway in the regulation of epidermal barrier function in AD.

In this review, we provide an update on the latest research efforts on the roles and mechanisms of PI3K/AKT/mTOR molecular axis in regulating hyperproliferative processes in the epidermal compartment of diseased skin. We also highlight on the current status of preclinical and clinical studies for the development of PI3K-targeted therapies in NMSC and psoriasis.

## Class I PI3K Enzyme Family and Key Intracellular Effectors

The phosphatidylinositol 3-kinases (PI3Ks) are members of a unique and conserved family of intracellular lipid kinases that phosphorylate the 3′-hydroxyl group of phosphatidylinositol and phosphoinositides (29). This reaction leads to the activation of many intracellular signaling pathways that regulate cell metabolism, survival, and vesicle trafficking.

Among PI3Ks enzymes, class I PI3Ks are the most widely characterized. These kinases show similar structure and share a common specificity for phosphatidyl inositol phosphates (PIPs) as substrates (2, 30–32).

Class I PI3Ks are divided into two subfamilies, named IA and IB, depending on their receptors. Indeed, class I PI3Ks are cytosolic enzymes in resting cells, and in response to different stimuli they are recruited to membranes by interacting with specific receptors or adaptor proteins (33, 34).

Class I PI3Ks are heterodimers that comprise a catalytic p110 subunit and a regulatory/adaptor subunit (35, 36). Class IA consists of one of the three catalytic isoforms p110 α, β, and δ and p85 regulatory subunit (p85α, β or their splice variants p55α, p50α, or p55γ), whereas class IB PI3K consists of p110γ catalytic isoform and p101 regulatory subunit (35). p110α and p110β are ubiquitously expressed and display distinct roles in cellular signaling, cell growth, angiogenesis, and oncogenic transformation (37–39). In contrast, PI3K p110δ is mainly expressed by hematopoietic cells and is critical for full B- and T-cell antigen receptor signaling (2, 40). PI3Kδ expression has also been reported in non-leucocyte cell types, such as breast cancer cells (41), neurons (42), lung and synovial fibroblasts, and endothelial cells (43, 44). We have recently observed PI3Kδ expression also in human keratinocytes and in the epidermis of a mouse skin inflammation model.

Mechanistically, the p85 regulatory subunit is crucial in mediating the activation of class IA PI3K by RTKs, through its direct binding to receptors on cell membranes. Upon receptor stimulation, p85 subunit recruits p110 to the intracellular phosphorylated tyrosine residues of RTKs, leading to p110 activation (45, 46). Activated PI3K p110 phosphorylates PIP2 to generate PIP3 that regulates multiple downstream pathways and cellular processes, such as membrane trafficking, cell growth, proliferation, metabolism, and migration (32, 47, 48). Once generated, PIP3 binds to several proteins, including AKT, also known as protein kinase B (PKB) (33, 45, 46).

AKT is a serine/threonine kinase that consists of three isoforms involved in numerous cellular processes, such as cell cycle progression, protein synthesis, glucose metabolism, cell proliferation, and survival (49–53). For a full activation of AKT, Thr308 and Ser473 residues located in two different domains need to be phosphorylated (46). Following PIP3 binding to AKT, this last is recruited to plasma membrane, where it is phosphorylated in Thr308 by phosphoinositide-dependent kinase-1 (PDK1) (49). One of the key elements of PI3K/AKT network is the serine/threonine kinase mammalian target of rapamycin (mTOR). mTOR can form two distinct multi-protein complexes, mTOR complex 1 (mTORC1) and mTOR complex 2 (mTORC2) (54). Following phosphorylation in Ser473 by mTORC2, AKT activates mTORC1 which in turn induces the phosphorylation of ribosomal protein S6 kinase beta-1 (S6K1), and eukaryotic translation initiation factor 4E-binding protein 1 (4E-BP1), a repressor of mRNA translation (55). As consequence, S6K1 phosphorylates S6 Ribosomal Protein (S6 Rb), whereas the inactivated 4E-BP1 repressor releases the eukaryotic translation initiation factor 4E (eIF4E) (55–57). Both phospho-S6Rb and eIF4E promote protein translation and cell proliferation (56). Of note, S6K1 can be phosphorylated also by PDK-1 without AKT involvement (58).

AKT not only regulates cell proliferation and protein synthesis, but also inhibits pro-apoptotic proteins, including BAD and caspase-9, two crucial pro-apoptotic components of cell death machinery, and forkhead box O (FOXO) transcription factor, a negative regulators of proliferation and cell survival (59–61). Moreover, AKT indirectly induces the transcription of anti-apoptotic genes via nuclear factor-κB (NF-κB) factors (62, 63).

Finally, PI3K-activated pathways play a key role in epidermal homeostasis by sustaining the proper epidermal formation, as well as keratinocyte differentiation and survival (64–67).

## Role of PI3K/AKT/MTOR Pathway in Growth and Proliferation in NMSC

**Figure 1:**
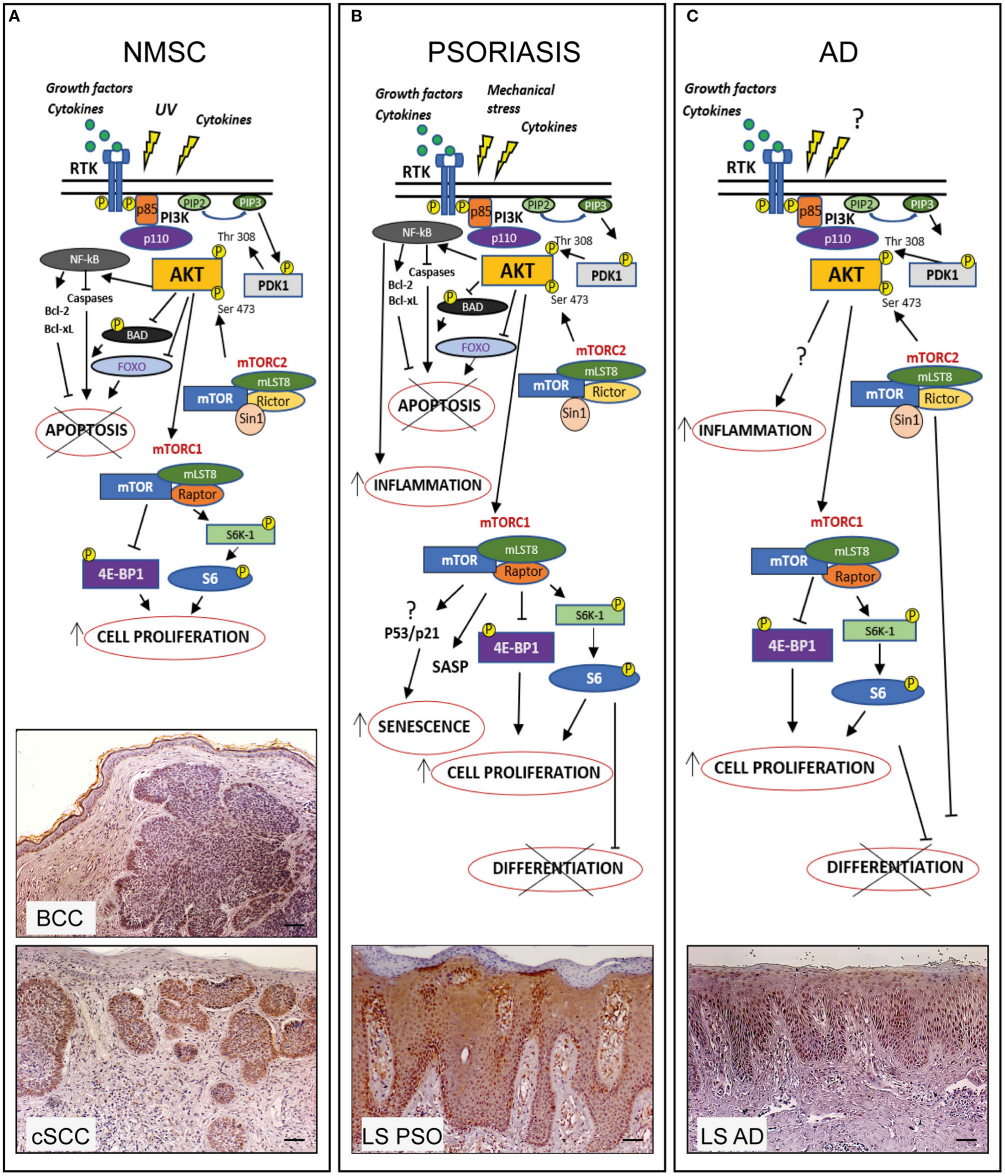
PI3K/AKT/mTOR pathway in hyperproliferative skin diseases. In healthy skin PI3K/AKT/mTOR pathway can be activated upon stimulation of receptor tyrosine kinases (RTK) leading to PI3K activation which in turn phosphorylates PIP2 to PIP3. Subsequently AKT is recruited to membrane and phosphorylated by PDK-1 and mTORC2. Phospho-AKT can induce mTORC1 activation by phosphorylating S6K-1 or 4E-BP1, thus controlling several cellular processes and maintaining the epidermal homeostasis. In hyperproliferative skin diseases as NMSC, psoriasis, and AD, several external stimuli are responsible for PI3K/AKT/mTOR over-expression and local increase of cytokines and growth factors lead to overexpression/upregulation of inflammatory molecular cascades contributing to progression of these skin disorders. In the three hyperproliferative skin conditions, PI3K/AKT/mTOR signaling is hyperactivated and involved in pathogenic processes (hyperproliferation, epidermal differentiation, inflammation, apoptosis, and senescence) depending on the disease context. The players of PI3K/AKT/mTOR pathway, as well as upstream/downstream mediators differentially activated in NMSC, psoriasis, and AD are schematically shown. Immunohistochemical analyses of p-AKT expression (red-brown color) of skin lesional areas of cSCC and BCC **(A)**, psoriatic plaques (LS PSO) **(B)**, and AD (LS AD) **(C)** show a wide expression of p-AKT in the epidermal layers of psoriasis and AD, as well as in cSCC and BCC, mostly expressed in tumor formations in both NMSCs types. Scale bars, 200 μm [Phospho-AKT stainings have been retrieved from Ref. (53, 73)].

Alterations in PI3K/AKT/mTOR signaling has been implicated in the pathogenesis and progression of numerous cutaneous cancers, including NMSC (68–70). In particular, hyperactivation of the PI3K/AKT axis has been detected in both SCC and BCC skin tissues, where suggesting its potential involvement in the pathogenesis and malignancy of these tumors (7, 71, 72) (Figure 1A; Table 1). Chen et al. found that the percentage of phosphorylated AKT (Ser473) positive cells is significantly higher in SCC than AK, and further enhanced in SCCs with metastases. AKT hyperactivation correlated with an increased phosphorylation of mTOR and downstream effectors, such as 4E-BP1, 70S6K1, p70S6K1, and S6 (Ser6) (76). Consistently, increased AKT activity is associated to nuclear accumulation of molecules involved in cell cycle progression, such as Cyclin D1, phosphorylated-c-myc, and β-catenin in cutaneous head and neck SCC (91).

**Table 1:** Expression and role of PI3K class IA p110 isoforms and downstream molecules in hyperproliferative skin diseases.

**Diseases**	**PI3Kα**	**PI3Kβ**	**PI3Kδ**	**p-AKT**	**p-mTOR**	**p-S6**	**p-4E-BP1**	**Role in pathogenesis**	**References**
BCC	+	+	?	+	+	++	?	Induction of proliferation Anti-apoptotic	(7, 72, 74, 75)
cSCC	++	++	?	++	+++	++	++	Induction of proliferation Anti-apoptotic	(7, 71, 75–78)
Psoriasis	+	+	++	+++	+++	++	++	Induction of proliferation Inhibition of epidermal differentiation Anti-apoptotic Pro-senescence Pro-inflammatory	(21, 53, 79–86)
AD	++	+	+	+++	?	?	?	Pro-inflammatory Inhibition of epidermal differentiation	(87–90)

*+/++/+++ symbols represent a scoring system to indicate the expression levels of PI3K/AKT/mTOR pathway components in BCC, cSCC, Psoriasis, and AD. +, ++, and +++ symbols indicate respectively weak, moderate, and high expression of the indicated molecules. Missing data on the expression of these molecule in literature are shown as the question mark “?”*.

Numerous reports have shown that PI3K/AKT/mTOR/S6K1 pathway can be activated in skin cancers by UV radiation exposure (54, 69, 70). In human and mouse epidermal keratinocytes, UV radiations induce the expression of p85 regulatory subunit and activates mTOR, with the consequent S6K1 phosphorylation (9, 92). UV radiation exposure can also determine the insurgence of mutations in PTEN gene, the major negative regulator of PI3Ks. PTEN commonly acts as a PI3K antagonist by dephosphorylating PIP3 to PIP2, and thus it inhibits AKT activation (93, 94). Recent evidence shows that chronic UVA radiation decreases PTEN expression, and this decrease is required for enhanced cell survival in transformed human keratinocytes, suggesting that PTEN might be critical for UVA-induced skin carcinogenesis (95). UVB was also reported to inhibit PTEN by increasing its stability and phosphorylation in human dermal fibroblasts (96). Thus, UV-mediated inhibition of PTEN further enhance AKT activation (92, 95, 96). Although a high frequency of PTEN mutations with consequent hyper-activation of AKT has been detected in malignant melanomas (97), the reduction of PTEN levels and the mechanism(s) by which its function and activity are regulated in NMSC remain to be established.

The mechanisms by which PI3K/AKT/mTOR pathway sustains NMSC development and progression involve both enhanced cell proliferation and resistance to apoptosis. In a recent study conducted on a transgenic mouse model developing multiple BCC, AKT1 isoform has been identified as obligatory for BCC tumorigenesis. Indeed, the pharmacological inhibition of AKT, as well as the genetic ablation of AKT1, diminished the growth of spontaneous and UV-induced tumors in this BCC murine model (74).

Previously, Zhao and colleagues investigated on the cutaneous expression of a series of pro-proliferative proteins, including AKT mediators, in transgenic mice expressing the tyrosine kinase Fyn, a model spontaneously forming keratotic lesions, scaly plaques, and large tumors, resembling AKs, carcinoma *in situ* (SCIS), and SCCs, respectively (98). They found increased levels of phosphorylated PDK1, together with STAT3 and ERK1/2, in both precancerous and SCCs lesions, compared with non-lesional epidermis. Of note, topical application of BEZ-235, a PI3K/mTOR inhibitor, induces regression of SCC in this disease model (77).

NMSC-infiltrating immune cells also indirectly contribute to tumor growth mediated by AKT pathway. Indeed, the release of pro-inflammatory cytokines, such as IL-22 by tumor-infiltrating lymphocytes (TILs) can promote *in vitro* keratinocyte hyperproliferation by sustaining AKT signaling the expression of cell cycle-related and anti-apoptotic molecules (99).

## Dual Effects of PI3K Pathways in Inflammatory and Hyperproliferative Skin Diseases

Hyperproliferation of epidermal keratinocytes contributes to the pathogenesis of several cutaneous disorders, including psoriasis and AD. Up-regulation of PI3K/AKT/mTOR pathway has been reported in skin of patients affected by psoriasis (Table 1), as well as in skin of imiquimod (IMQ)-induced psoriasiform mouse model (53, 79). In fact, Pike and colleagues measured a higher PI3K activity in epidermis of psoriatic patients than in healthy donors (80). Consistently, our group reported a strong expression of phosphorylated AKT in lesional psoriatic skin and in cytokine-activated keratinocytes derived from patients affected by psoriasis (53). Expression of the PI3K effectors phospho-AKT, -S6K1, -S6 Rb, and 4E-BP1, is more pronounced in suprabasal keratinocytes, whereas mTOR is hyperactivated in all epidermal layers of lesional psoriatic skin (53, 81). The wide expression of mTOR in psoriatic epidermis may be associated not only to a keratinocyte hyperproliferation (81, 82, 100), but also to aberrant differentiation, since AKT/mTOR pathway inactivation is requested during keratinocytes terminal differentiation (21, 101) (Figure 1B). In contrast, reduced levels of the FOXO1 and PTEN regulators have been observed in psoriatic epidermis (102–105).

AKT can also prevent cytokine-induced cellular apoptosis and promote senescence-like growth arrest in psoriasis (53) (Figure 1B). Indeed, psoriatic keratinocytes exhibit a senescent phenotype characterized by a peculiar resistance to apoptosis, secretion of inflammatory molecules, and expression of specific markers of senescence, which contributes to the epidermal thickening typically observed in psoriatic skin (106–108). Interestingly, the chemical inhibition of PI3K/AKT cascade by Ly294002 molecule renders psoriatic keratinocytes more susceptible to pro-apoptotic stimuli, such as pro-inflammatory Th1/17-released cytokines (53). However, the mechanism(s) by which PI3K/AKT axis sustains senescence phenotype in psoriatic keratinocytes remains to be established.

In support of our observation, Miyauchi et al. have reported that AKT promotes a senescence-like phenotype also in endothelial cells via tumor suppressor TP53 (P53) and cyclin-dependent kinase inhibitor p21^WAF1/Cip1^ (p21)-dependent pathway (109). In particular, constitutive activation of Akt inhibits the transcriptional activity of FOXO3a and thereby downregulates manganese superoxide dismutase, leading to an increase of ROS that promotes senescence-like growth arrest by inducing p53 activity and p21 expression (106). Additionally, chronic hyperactivation of AKT in human non-transformed fibroblasts results in a TORC1-dependent increase in p53 translation, and simultaneously stimulates MDM2 sequestration within the nucleous, thus inhibiting p53 ubiquitination and degradation. This event results in an accumulation of p53, leading to cellular senescence (110, 111). Finally, in a recent genome-wide RNAi screening study, three novel intracellular mediators of senescence induced by AKT have been identified in human fibroblasts, including the pro-apoptotic CCAR1 and FADD proteins, and NF1, the negative regulator of RAS/ERK signaling (112). Based on these data, we hypothesize that the hyperactivation PI3K/AKT pathway in psoriatic lesions could be implicated in the regulation of the senescent-like phenotype of epidermal keratinocytes.

The role of PI3K/AKT/mTOR in AD is less characterized than in psoriasis, even though some evidences have accumulated so far (Figure 1C; Table 1). Topical application of the mTOR inhibitor, rapamycin, in experimental models of AD induced by different antigens in NC/Nga mice improves several clinical parameters, including epidermal thickness, dermal inflammatory infiltrate, serum IgE and Th2 and Th1 cytokine levels (87, 88).

Recent evidence has demonstrated the mTOR role in epidermal stratification and cornification. Indeed, Ding and colleagues, have shown that mTORC2 controls FLG processing and *de novo* epidermal lipid synthesis during cornification in mice lacking RICTOR in the epidermis (113). Furthermore, increased transcriptional levels of the regulatory-associated protein of mTORC1, RAPTOR, correlate with decreased FLG expression, barrier defects and presence of inflammatory markers in skin of patients with AD (114). Of interest, it has been recently reported that Th2-released IL-13 could activate the mTOR signaling pathway in human immortalized keratinocytes, and the pharmacological inhibition of mTORC1 by rapamycin blocks the IL-13-induced expression of p-mTOR, p-S6K1, and p-AKT. Concomitantly, in human keratinocytes rapamycin up-regulates the expression of terminal differentiation markers, including filaggrin, loricrin, and involucrin, typically impaired in AD skin lesions (89).

Finally, PI3K/AKT signaling is abnormally activated in peripheral T cells from pediatric AD patients. Of note, the PI3K inhibitor LY294002 significantly inhibits proliferation and release of the IL-10 and IL-6 cytokines in AD patient-derived T cells, thus suggesting a relevant role of PI3K pathways in AD inflammatory circuits (90). All these data suggest that PI3K/AKT blocking could be a potential effective therapeutic option in the management of AD.

## PI3K Therapeutic Targeting in Hyperproliferative Skin Disorders

In last years, targeting PI3K/AKT/mTOR axis proved to be a promising tool for treatment of NMSC, especially mTOR targeting by specific inhibitors. In BCC patients, treatment with the mTORC1 inhibitor Everolimus leads to a partial or complete tumor recession (70, 115–117). Furthermore, Itraconazole, previously discovered as an antifungal agent, has been found to have anticancer action by inhibiting mTOR signaling (118), and a recent clinical trial conducted in BCC patients have shown encouraging effectiveness (119). Other mTORC1 inhibitors, such as rapamycin and its analogs, showed a better clinical response in cSCC than in BCC, probably due to the higher mTOR expression in SCC epidermal tissue (70, 75, 78, 120).

However, the first generation mTOR inhibitors, selective for mTORC1 and with a poor action on mTORC2, led to a subsequent AKT activation. Thus, a second generation of mTOR inhibitors, targeting both mTORC1 and mTORC2, have been developed (121). Among these, GDC-0084 exhibited a potent anti-proliferative effect on cSCC in preclinical studies (122). LY3023414, a small PI3K-AKT dual inhibitor, showed a strong cytotoxic and anti-proliferative effect on SCC cell lines and in tumor xenografts models, and it is currently used in phase I and II clinical trials (123).

Of note, PI3K/AKT/mTOR signaling has been described to be involved in resistance to specific inhibitors classically employed in NMSCs, due to the intricate crosstalk between different pathways in these skin cancers (70, 117). Thus, the use of PI3K/AKT/mTOR inhibitors in combination with agents targeting other pathways is more effective in contrasting drug resistance (124).

In psoriasis, rapamycin (Sirolimus) has been employed via oral administration, alone and combination with cyclosporine. Despite enhanced ameliorative effects with the two drugs combined, rapamycin alone was ineffective (125). In contrast, a clinical trial with topical application of Sirolimus in psoriatic patients reported a decrease in clinical score, together with a significant reduction in CD4-positive T cells and proliferating Ki67^+^ cells in the epidermis. However, no effects on plaque thickness and erythema have been observed (126). These data suggest that mTOR inhibition does not exert significant improvement in psoriasis.

PI3K isoforms has been instead described as efficacious targets in treating psoriasis. PI3K inhibition results in reduction of epidermal thickness, number of infiltrating immune cells and levels of psoriasis-related cytokines in the IMQ-induced psoriasiform mouse model (83, 84). In addition, blocking of PI3Ks counteracts proliferation and activation processes in T cells derived from psoriatic patients (84). The selective PI3kδ inhibitor Seletalisib can reduce *in vitro* production of pro-inflammatory cytokines from IL-17-producing adaptive and innate-like lymphocytes (85, 86). Consistently, a recent first-in-human study of oral administration of the PI3Kδ inhibitor Seletalisib showed ameliorative effects on size and appearance of psoriatic lesions, together with a reduction in T cells and neutrophils, in skin from psoriasis patients undergone Seletalisib treatment (127). In line with these data, we observed that the topical administration of Seletalisib drastically reduced epidermal thickening and the number of infiltrating neutrophils in an IMQ-induced psoriasiform murine model. These findings support the clinical development of PI3K p110δ isoform inhibitors in psoriasis.

In AD, PI3K/AKT/mTOR inhibitors have not been yet tested. PI3K p110δ has been shown to be involved in type-2 inflammation associated to atopy/allergy (128), and, in particular, in the development of Th2 asthma, a common pathological symptom of many allergic diseases, including AD. In support of this, the selective inhibition of PI3K p110 δ and γ isoforms resulted in the attenuation of allergic airway inflammation in several preclinical models (129–133).

Therefore, blocking PI3K/AKT/mTOR could be an effective therapeutic strategy in AD treatment, being this pathway involved in the pathogenic mechanisms resulting in AD symptoms, as defective epidermal barrier, inflammation and allergic asthma. However, further investigations are needed to better understand the impact of PI3K/AKT/mTOR inhibition in AD clinical resolution.

## Conclusions

PI3K/AKT pathway is implicated in NMSC development and progression, as well as in the pathogenic mechanisms associated to chronic inflammatory skin conditions, such as psoriasis and AD. However, our understanding of this complex network and its tight regulation is at its beginning and will need much more work to definitively assess the impact of its inhibition in the clinical outcomes of these hyperproliferative skin disorders. Targeting PI3K/AKT pathway in NMSCs, with synthetic small molecules alone or in various combinations, have been widely employed in clinical trials with effective clinical response, although several of these agents display limitations as undesired side effects. Thus, a careful selection and development of more potent and safer agents are needed. Moreover, a more in-depth characterization of the role of distinct PI3K isoforms in NMSCs are needed to determine whether targeting selective PI3Ks could represent a powerful strategy to counteract these diseases.

In the context of inflammatory skin condition, despite recent drug development has mainly centered on biological therapies for psoriasis and AD management, small molecule drugs targeting specific PI3K isoforms or combined drugs acting on multiple PI3K effectors, administrated orally or topically, could represent a valid alternative for treating psoriasis or AD patients undergone clinical failure with biologics or psoriasis patients affected by challenging-to-treat clinical subtypes.

## Author Contributions

All authors have contributed in the ideation and writing of the manuscript and checked the final version of the paper.

## Conflict of Interest

The authors declare that the research was conducted in the absence of any commercial or financial relationships that could be construed as a potential conflict of interest.
